# (*N* ^1^ *E*,*N* ^2^ *E*)-*N* ^1^,*N* ^2^-Bis(4-hex­yloxy-3-methoxy­benzyl­idene)ethane-1,2-diamine

**DOI:** 10.1107/S1600536810017125

**Published:** 2010-05-19

**Authors:** Anju Paul, Sherin Susan Punnoose, N. L. Mary, T. Narasimhaswamy, V. Ramkumar

**Affiliations:** a Department of Chemistry, Stella Maris College, Chennai, TamilNadu, India; b Polymer Division, C.L.R.I. Adyar, Chennai, TamilNadu, India; c Department of Chemistry, IIT Madras, Chennai, TamilNadu, India

## Abstract

The title compound, C_30_H_44_N_2_O_4_, was obtained from the dimerization of 4-hexyl­oxyvanillin with ethyl­enediamine in 95% methanol solution. It adopts a *trans* configuration with respect to the C=N bond and possesses a crystallographically imposed centre of symmetry.

## Related literature

For Schiff bases derived from vanillin, see: Guo *et al.* (2008 ▶); Li (2008 ▶). For its biological activity, see: Liang *et al.* (2009 ▶); Lim *et al.* (2008 ▶). For the potential uses of mol­ecular materials with supra­molecular architectures in emerging technologies and medicine, see: Porta *et al.* (2008 ▶). For details of the preparation of the title compound, see: Dholakiya & Patel (2002 ▶); Maurya *et al.* (2003 ▶); Doyle *et al.* (2007 ▶).
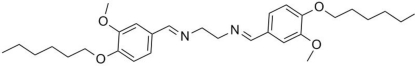


## Experimental

### 

#### Crystal data

C_30_H_44_N_2_O_4_
*M* *_r_* = 496.67
Triclinic, 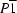

*a* = 5.3025 (6) Å
*b* = 10.3777 (14) Å
*c* = 13.0463 (17) Å
α = 84.667 (6)°
β = 84.659 (6)°
γ = 89.045 (6)°
*V* = 711.67 (16) Å^3^
*Z* = 1
Mo *K*α radiation
μ = 0.08 mm^−1^
*T* = 298 K
0.45 × 0.22 × 0.10 mm

#### Data collection

Bruker APEXII CCD area-detector diffractometer
Absorption correction: multi-scan (*SADABS*; Bruker, 2004 ▶) *T*_min_ = 0.967, *T*_max_ = 0.992
9758 measured reflections
3249 independent reflections
1873 reflections with *I* > 2σ(*I*)
*R*_int_ = 0.023

#### Refinement

*R*[*F* ^2^ > 2σ(*F* ^2^)] = 0.053
*wR*(*F* ^2^) = 0.167
*S* = 1.03
3249 reflections
165 parameters
H-atom parameters constrained
Δρ_max_ = 0.22 e Å^−3^
Δρ_min_ = −0.23 e Å^−3^

### 

Data collection: *APEX2* (Bruker, 2004 ▶); cell refinement: *APEX2* and *SAINT-Plus* (Bruker, 2004 ▶); data reduction: *SAINT-Plus* and *XPREP* (Bruker, 2004 ▶); program(s) used to solve structure: *SHELXS97* (Sheldrick, 2008 ▶); program(s) used to refine structure: *SHELXL97* (Sheldrick, 2008 ▶); molecular graphics: *ORTEP-3* (Farrugia, 1997 ▶); software used to prepare material for publication: *SHELXL97*.

## Supplementary Material

Crystal structure: contains datablocks global, I. DOI: 10.1107/S1600536810017125/jh2151sup1.cif

Structure factors: contains datablocks I. DOI: 10.1107/S1600536810017125/jh2151Isup2.hkl

Additional supplementary materials:  crystallographic information; 3D view; checkCIF report

## Figures and Tables

**Fig. 1.**
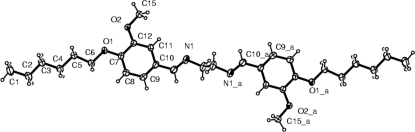
ORTEP of the molecule with atoms represented as 30% probability ellipsoids.

## supplementary crystallographic information

### Comment

The title compound C_30_H_44_N_2_O_4_ is a synthetic analogue with a long aliphatic side chain of vanillin. The Schiff base derived from vanillin (Guo *et.al*, 2008; Li *et.al*, 2008) exhibit potential antibacterial activity and a potent anti-proliferative effect on a broad spectrum of cancer cell lines (Liang. *et.al*, 2009; Lim *et.al*, 2008).

The design of synthetic molecules with self-organised behaviour is one of the fastest growing areas of research. Molecular materials that arise from the self organising properties of the molecules may afford supramolecular architectures (structures beyond the molecule) with chemical and physical properties that may become useful in emerging technologies and medicine (Porta *et.al*, 2008). Molecules that use non-covalent interactions to self-organise into supramolecular structures have the potential to generate functional materials with a broad range of applications. This unique combination of coordination bond and alkyl interdigitation provide exceptional control over intermolecular interactions and can generate nano scale molecular order as liquid crystalline states and Langmiur-Blodgett films on surfaces.

The crystal adopts a *trans* configuration with respect to the C=N bond and possesses a crystallographically imposed centre of symmetry.

### Experimental

1) Synthesis of 4-hexyloxy vanillin.

15.215g (0.1 mole) of vanillin was dissolved in 300ml of dimethylformamide in a round-bottom flask. 17.96g (0.13 mole) of potassium carbonate was also added. The resulting mixture was stirred by using a homogeniser maintaining the temperature at 90° C by using an oil bath. 14.03ml (0.1 mole) of bromohexane was added to the reaction mixture through a dropping funnel over a period of 30 minutes (Dholakiya *et.al*, 2002; Maurya *et.al*, 2003). The resulting mixture was stirred for 3 hours and cooled to room temperature, diluted with 600ml water. The contents were transferred to a separating funnel extracted with diethyl ether, washed with 5% KOH solution and water respectively. 4-hexyloxy vanillin was obtained and it was recrystallised from hot alcoholic solution.

2) Dimerisation of 4-hexyloxy vanillin with ethylenediamine.

3g (0.05 mole) of ethylenediamine was dissolved in 10ml of ethanol in a round-bottom flask. 23.6g (0.1 mole) of 4-hexyloxy vanillin and 5 drops of acetic acid were added into it. It was fitted to a water condenser and heated for 2 hours (Doyle *et.al*, 2007). It was allowed to cool, washed with methanol and dried in an oven. Recrystallisation of the compound from methanol gave X-ray diffraction quality crystals of the title compound.

### Refinement

All hydrogen atoms were fixed geometrically and allowed to ride on the parent carbon atoms with aromatic C-H = 0.93 Å, aliphatic C-H = 0.98 Å and methyl C-H = 0.96 Å. The displacement parameters were set for phenyl and aliphatic H atoms at *U*_iso_(H) = 1.2*U*_eq_(C) and for methyl H atoms at *U*_iso_(H) = 1.5*U*_eq_(C)

### Crystal data

**Table:**

C_30_H_44_N_2_O_4_	*Z* = 1
*M**_r_* = 496.67	*F*(000) = 270
Triclinic, *P*1	*D*_x_ = 1.159 Mg m^−^^3^
Hall symbol: -P 1	Mo *K*α radiation, λ = 0.71073 Å
*a* = 5.3025 (6) Å	Cell parameters from 2606 reflections
*b* = 10.3777 (14) Å	θ = 2.5–24.8°
*c* = 13.0463 (17) Å	µ = 0.08 mm^−^^1^
α = 84.667 (6)°	*T* = 298 K
β = 84.659 (6)°	Rectangular, colourless
γ = 89.045 (6)°	0.45 × 0.22 × 0.10 mm
*V* = 711.67 (16) Å^3^	

### Data collection

**Table:**

Bruker APEXII CCD area-detector diffractometer	3249 independent reflections
Radiation source: fine-focus sealed tube	1873 reflections with *I* > 2σ(*I*)
graphite	*R*_int_ = 0.023
phi and ω scans	θ_max_ = 28.3°, θ_min_ = 2.0°
Absorption correction: multi-scan (*SADABS*; Bruker, 2004)	*h* = −7→5
*T*_min_ = 0.967, *T*_max_ = 0.992	*k* = −13→13
9758 measured reflections	*l* = −17→17

### Refinement

**Table:**

Refinement on *F*^2^	Primary atom site location: structure-invariant direct methods
Least-squares matrix: full	Secondary atom site location: difference Fourier map
*R*[*F*^2^ > 2σ(*F*^2^)] = 0.053	Hydrogen site location: inferred from neighbouring sites
*wR*(*F*^2^) = 0.167	H-atom parameters constrained
*S* = 1.03	*w* = 1/[σ^2^(*F*_o_^2^) + (0.0813*P*)^2^ + 0.0648*P*] where *P* = (*F*_o_^2^ + 2*F*_c_^2^)/3
3249 reflections	(Δ/σ)_max_ < 0.001
165 parameters	Δρ_max_ = 0.22 e Å^−^^3^
0 restraints	Δρ_min_ = −0.23 e Å^−^^3^

### Special details

**Table:**

Geometry. All esds (except the esd in the dihedral angle between two l.s. planes) are estimated using the full covariance matrix. The cell esds are taken into account individually in the estimation of esds in distances, angles and torsion angles; correlations between esds in cell parameters are only used when they are defined by crystal symmetry. An approximate (isotropic) treatment of cell esds is used for estimating esds involving l.s. planes.
Refinement. Refinement of F^2^ against ALL reflections. The weighted R-factor wR and goodness of fit S are based on F^2^, conventional R-factors R are based on F, with F set to zero for negative F^2^. The threshold expression of F^2^ > 2sigma(F^2^) is used only for calculating R-factors(gt) etc. and is not relevant to the choice of reflections for refinement. R-factors based on F^2^ are statistically about twice as large as those based on F, and R- factors based on ALL data will be even larger.

### Fractional atomic coordinates and isotropic or equivalent isotropic displacement parameters (Å^2^)

**Table:**

	*x*	*y*	*z*	*U*_iso_*/*U*_eq_	
C1	1.3152 (4)	0.4143 (2)	0.8601 (2)	0.0941 (7)	
H1A	1.4109	0.4622	0.9025	0.141*	
H1B	1.4280	0.3615	0.8196	0.141*	
H1C	1.1958	0.3602	0.9034	0.141*	
C2	1.1755 (4)	0.50694 (19)	0.78939 (16)	0.0719 (6)	
H2A	1.2985	0.5568	0.7428	0.086*	
H2B	1.0780	0.4574	0.7477	0.086*	
C3	0.9998 (3)	0.59913 (17)	0.84379 (14)	0.0583 (5)	
H3A	1.0965	0.6484	0.8860	0.070*	
H3B	0.8748	0.5497	0.8896	0.070*	
C4	0.8650 (3)	0.69171 (18)	0.77122 (14)	0.0581 (5)	
H4A	0.7761	0.6418	0.7268	0.070*	
H4B	0.9911	0.7430	0.7274	0.070*	
C5	0.6765 (3)	0.78325 (16)	0.82227 (13)	0.0521 (4)	
H5A	0.5492	0.7336	0.8667	0.063*	
H5B	0.7635	0.8366	0.8648	0.063*	
C6	0.5513 (3)	0.86768 (16)	0.74259 (13)	0.0515 (4)	
H6A	0.6769	0.9218	0.7011	0.062*	
H6B	0.4736	0.8144	0.6971	0.062*	
C7	0.2265 (3)	1.02721 (15)	0.72825 (12)	0.0446 (4)	
C8	0.2641 (3)	1.03991 (17)	0.62159 (12)	0.0555 (5)	
H8	0.3923	0.9928	0.5882	0.067*	
C9	0.1123 (3)	1.12212 (18)	0.56439 (13)	0.0591 (5)	
H9	0.1388	1.1290	0.4926	0.071*	
C10	−0.0761 (3)	1.19360 (15)	0.61098 (12)	0.0487 (4)	
C11	−0.1152 (3)	1.18130 (15)	0.71918 (12)	0.0494 (4)	
H11	−0.2432	1.2293	0.7520	0.059*	
C12	0.0324 (3)	1.09964 (14)	0.77722 (11)	0.0447 (4)	
C13	−0.2366 (4)	1.27834 (18)	0.54763 (14)	0.0602 (5)	
H13	−0.2200	1.2727	0.4766	0.072*	
C14	−0.5432 (4)	1.43145 (18)	0.50776 (15)	0.0694 (6)	
H14A	−0.7211	1.4286	0.5331	0.083*	
H14B	−0.5236	1.3933	0.4424	0.083*	
C15	−0.2111 (4)	1.13500 (19)	0.93525 (13)	0.0670 (5)	
H15A	−0.1983	1.2276	0.9260	0.101*	
H15B	−0.2181	1.1066	1.0076	0.101*	
H15C	−0.3621	1.1088	0.9078	0.101*	
N1	−0.3933 (3)	1.35719 (15)	0.58257 (12)	0.0671 (5)	
O1	0.3626 (2)	0.94721 (10)	0.79130 (8)	0.0519 (3)	
O2	0.0049 (2)	1.07846 (12)	0.88225 (8)	0.0605 (4)	

### Atomic displacement parameters (Å^2^)

**Table:**

	*U*^11^	*U*^22^	*U*^33^	*U*^12^	*U*^13^	*U*^23^
C1	0.0811 (16)	0.0828 (16)	0.1147 (19)	0.0396 (13)	−0.0043 (14)	−0.0015 (14)
C2	0.0653 (12)	0.0674 (12)	0.0814 (13)	0.0241 (10)	0.0002 (10)	−0.0095 (10)
C3	0.0479 (10)	0.0593 (11)	0.0672 (11)	0.0142 (8)	−0.0050 (8)	−0.0067 (9)
C4	0.0467 (10)	0.0634 (11)	0.0629 (11)	0.0134 (9)	−0.0027 (8)	−0.0038 (9)
C5	0.0451 (9)	0.0534 (10)	0.0577 (10)	0.0132 (8)	−0.0068 (7)	−0.0043 (8)
C6	0.0430 (9)	0.0546 (10)	0.0564 (10)	0.0115 (8)	−0.0023 (7)	−0.0076 (8)
C7	0.0454 (9)	0.0438 (9)	0.0441 (9)	0.0079 (7)	−0.0086 (7)	0.0017 (7)
C8	0.0561 (10)	0.0636 (11)	0.0444 (9)	0.0144 (9)	0.0013 (8)	−0.0004 (8)
C9	0.0700 (12)	0.0654 (11)	0.0395 (9)	0.0083 (10)	−0.0055 (8)	0.0058 (8)
C10	0.0595 (11)	0.0427 (9)	0.0442 (9)	0.0042 (8)	−0.0163 (7)	0.0047 (7)
C11	0.0590 (10)	0.0427 (9)	0.0472 (9)	0.0153 (8)	−0.0132 (8)	−0.0016 (7)
C12	0.0539 (10)	0.0414 (8)	0.0388 (8)	0.0091 (8)	−0.0094 (7)	−0.0007 (6)
C13	0.0760 (13)	0.0563 (11)	0.0489 (10)	0.0035 (10)	−0.0167 (9)	0.0026 (8)
C14	0.0782 (14)	0.0599 (11)	0.0704 (12)	0.0094 (10)	−0.0297 (10)	0.0136 (9)
C15	0.0789 (13)	0.0752 (13)	0.0447 (9)	0.0331 (10)	−0.0010 (9)	−0.0037 (8)
N1	0.0792 (11)	0.0629 (10)	0.0585 (9)	0.0163 (9)	−0.0208 (8)	0.0088 (8)
O1	0.0519 (7)	0.0569 (7)	0.0461 (6)	0.0233 (6)	−0.0072 (5)	−0.0009 (5)
O2	0.0727 (8)	0.0701 (8)	0.0373 (6)	0.0373 (6)	−0.0073 (5)	−0.0017 (5)

### Geometric parameters (Å, °)

**Table:**

C1—C2	1.504 (3)	C7—C12	1.405 (2)
C1—H1A	0.9600	C8—C9	1.380 (2)
C1—H1B	0.9600	C8—H8	0.9300
C1—H1C	0.9600	C9—C10	1.365 (2)
C2—C3	1.503 (2)	C9—H9	0.9300
C2—H2A	0.9700	C10—C11	1.402 (2)
C2—H2B	0.9700	C10—C13	1.467 (2)
C3—C4	1.505 (3)	C11—C12	1.369 (2)
C3—H3A	0.9700	C11—H11	0.9300
C3—H3B	0.9700	C12—O2	1.3620 (18)
C4—C5	1.519 (2)	C13—N1	1.243 (2)
C4—H4A	0.9700	C13—H13	0.9300
C4—H4B	0.9700	C14—N1	1.470 (2)
C5—C6	1.493 (2)	C14—C14^i^	1.492 (4)
C5—H5A	0.9700	C14—H14A	0.9700
C5—H5B	0.9700	C14—H14B	0.9700
C6—O1	1.4274 (18)	C15—O2	1.428 (2)
C6—H6A	0.9700	C15—H15A	0.9600
C6—H6B	0.9700	C15—H15B	0.9600
C7—O1	1.3602 (18)	C15—H15C	0.9600
C7—C8	1.382 (2)		
			
C2—C1—H1A	109.5	O1—C7—C8	124.79 (15)
C2—C1—H1B	109.5	O1—C7—C12	116.27 (13)
H1A—C1—H1B	109.5	C8—C7—C12	118.93 (14)
C2—C1—H1C	109.5	C9—C8—C7	120.33 (16)
H1A—C1—H1C	109.5	C9—C8—H8	119.8
H1B—C1—H1C	109.5	C7—C8—H8	119.8
C3—C2—C1	114.57 (19)	C10—C9—C8	121.35 (15)
C3—C2—H2A	108.6	C10—C9—H9	119.3
C1—C2—H2A	108.6	C8—C9—H9	119.3
C3—C2—H2B	108.6	C9—C10—C11	118.64 (15)
C1—C2—H2B	108.6	C9—C10—C13	119.84 (15)
H2A—C2—H2B	107.6	C11—C10—C13	121.51 (16)
C2—C3—C4	113.47 (16)	C12—C11—C10	120.86 (15)
C2—C3—H3A	108.9	C12—C11—H11	119.6
C4—C3—H3A	108.9	C10—C11—H11	119.6
C2—C3—H3B	108.9	O2—C12—C11	125.23 (14)
C4—C3—H3B	108.9	O2—C12—C7	114.87 (13)
H3A—C3—H3B	107.7	C11—C12—C7	119.88 (14)
C3—C4—C5	115.64 (15)	N1—C13—C10	124.35 (17)
C3—C4—H4A	108.4	N1—C13—H13	117.8
C5—C4—H4A	108.4	C10—C13—H13	117.8
C3—C4—H4B	108.4	N1—C14—C14^i^	109.91 (19)
C5—C4—H4B	108.4	N1—C14—H14A	109.7
H4A—C4—H4B	107.4	C14^i^—C14—H14A	109.7
C6—C5—C4	110.57 (14)	N1—C14—H14B	109.7
C6—C5—H5A	109.5	C14^i^—C14—H14B	109.7
C4—C5—H5A	109.5	H14A—C14—H14B	108.2
C6—C5—H5B	109.5	O2—C15—H15A	109.5
C4—C5—H5B	109.5	O2—C15—H15B	109.5
H5A—C5—H5B	108.1	H15A—C15—H15B	109.5
O1—C6—C5	110.11 (13)	O2—C15—H15C	109.5
O1—C6—H6A	109.6	H15A—C15—H15C	109.5
C5—C6—H6A	109.6	H15B—C15—H15C	109.5
O1—C6—H6B	109.6	C13—N1—C14	116.83 (17)
C5—C6—H6B	109.6	C7—O1—C6	116.96 (12)
H6A—C6—H6B	108.2	C12—O2—C15	117.33 (12)
			
C1—C2—C3—C4	179.30 (18)	O1—C7—C12—O2	−0.7 (2)
C2—C3—C4—C5	177.47 (15)	C8—C7—C12—O2	178.37 (15)
C3—C4—C5—C6	−178.66 (15)	O1—C7—C12—C11	−179.24 (14)
C4—C5—C6—O1	176.14 (13)	C8—C7—C12—C11	−0.2 (2)
O1—C7—C8—C9	178.75 (15)	C9—C10—C13—N1	−171.75 (17)
C12—C7—C8—C9	−0.2 (3)	C11—C10—C13—N1	9.9 (3)
C7—C8—C9—C10	0.6 (3)	C10—C13—N1—C14	−178.23 (15)
C8—C9—C10—C11	−0.6 (3)	C14^i^—C14—N1—C13	−107.7 (3)
C8—C9—C10—C13	−178.96 (16)	C8—C7—O1—C6	−2.4 (2)
C9—C10—C11—C12	0.1 (2)	C12—C7—O1—C6	176.64 (13)
C13—C10—C11—C12	178.52 (15)	C5—C6—O1—C7	−178.19 (13)
C10—C11—C12—O2	−178.15 (15)	C11—C12—O2—C15	7.1 (2)
C10—C11—C12—C7	0.2 (2)	C7—C12—O2—C15	−171.33 (15)

Symmetry codes: (i) −*x*−1, −*y*+3, −*z*+1.
